# Lack of cold temperatures is driving recent high-summer warming in the southern Rocky Mountains

**DOI:** 10.1007/s00484-025-02904-9

**Published:** 2025-03-31

**Authors:** M. C. A. Torbenson, E. Martinez del Castillo, F. Reinig, D. W. Stahle, K. E. King, J. T. Maxwell, G. L. Harley, E. Ziaco, J. Esper

**Affiliations:** 1 Department of Geography, Johannes Gutenberg University, Mainz, Germany; 2 Global Change Research Institute, Czech Academy of Sciences, Brno, Czech Republic; 3 Department of Geosciences, University of Arkansas, Fayetteville, AR USA; 4 Department of Geography and Sustainability, University of Tennessee, Knoxville, TN USA; 5 Department of Geography, Indiana University, Bloomington, IN USA; 6 Department of Earth and Spatial Sciences, University of Idaho, Moscow, ID USA

**Keywords:** Warming, Temperature variability, Dendroclimatology, Reconstruction, Long-term trend

## Abstract

We present a 483-year-long tree-ring chronology from a high-elevation Engelmann spruce stand in central Colorado. Over 800,000 density measurements produce a maximum latewood density (MXD) record, which together with tree-ring widths, is used to assess the climatic influences on radial tree growth. Variability in MXD is strongly related to local and regional August maximum temperatures (T_max_) for 127 years of overlapping instrumental climate data. A single-predictor reconstruction based on the MXD record explains > 50% of the variance in regional August T_max_ is presented and considered robust for the period 1662–2021. The estimates of past temperature variability display phases of continuous cold conditions that are beyond the range of the instrumental record, including the 1830s. Our results further indicate that August T_max_ have been higher since the early 1990s than for any other 30-year period in the past 350 years. This recent increase occurs on top of multi-centennial warming, and appears to be driven mainly by less frequent cold summers rather than an increase in warm extremes.

**Supplementary Information:**

The online version contains supplementary material available at 10.1007/s00484-025-02904-9.

## Introduction

Increasing temperatures are having profound ecological and societal impacts in western North America (Overpeck and Udall 2020; Williams et al. 2020; Hagmann et al. 2021). Yet trends and changes in temperature may differ considerably across space and seasons (Pielke et al. 2002). Temperatures are thought to have risen more rapidly at high altitudes globally (Wang et al. 2013), but such assertions are hampered by poor coverage of instrumental observations (Rangwala and Miller 2012). In the western United States, greater rates of warming at high elevations have been put into question as they may stem from analytical artifacts such as differences in or change to observation protocols and instrumentation (Oyler et al. 2015). Putting these already ambiguous trends into long-term contexts, beyond the relatively short instrumental records of climate variability, is fraught with uncertainty (Pepin et al. 2022). Our understanding of current temperature variability and change in mountainous regions, including the Rocky Mountains, therefore benefits from alternative sources of information, such as proxy records.

Tree rings have been used extensively to produce estimates of temperature variability hundreds, and sometimes thousands, of years back in time (e.g., D’Arrigo et al. 2006; Esper et al. 2016; Anchukaitis et al. 2017). Many such reconstructions rely on total-ring width (TRW), but parameters derived from wood density of the individual growth rings, such as maximum latewood density (MXD), can record stronger temperature signals compared to that of TRW (Hartl et al. 2022). In North America, a large network of tree-ring density measurements was developed in the 1980s and represents the foundation of our understanding of regional summer temperature variability prior to instrumental observations (Briffa et al. 1992, 1994). However, although alternatives have been developed (Heeter et al. 2020), few MXD records from the region have been produced since then and the recent warming experienced in western North America (Meehl et al. 2012; Thompson et al. 2022) is not covered by any of these early density-based proxy records. The lack of updates to important MXD records represents an example of a long-standing problem in dendroclimatology that has been highlighted for over a decade (Larson et al. 2013).

Here we present a new collection of density measurements of Engelmann spruce (*Picea engelmannii* Parry ex. Engelman) from the southern Front Range in the state of Colorado – a site part of the 1980s density network of Briffa et al. (1992). We assess if, and how, the temperature signal in the MXD record has changed in the 38 years that followed the original sampling. Our tree-ring record tracks instrumental temperature variability across the full temporal spectrum during the 20th and early twenty-first centuries, and we therefore argue that the subsequent reconstruction provides robust estimates of high-summer maximum temperature for the past 400 years.

## Materials and methods

### Tree-ring data

**Fig. 1:**
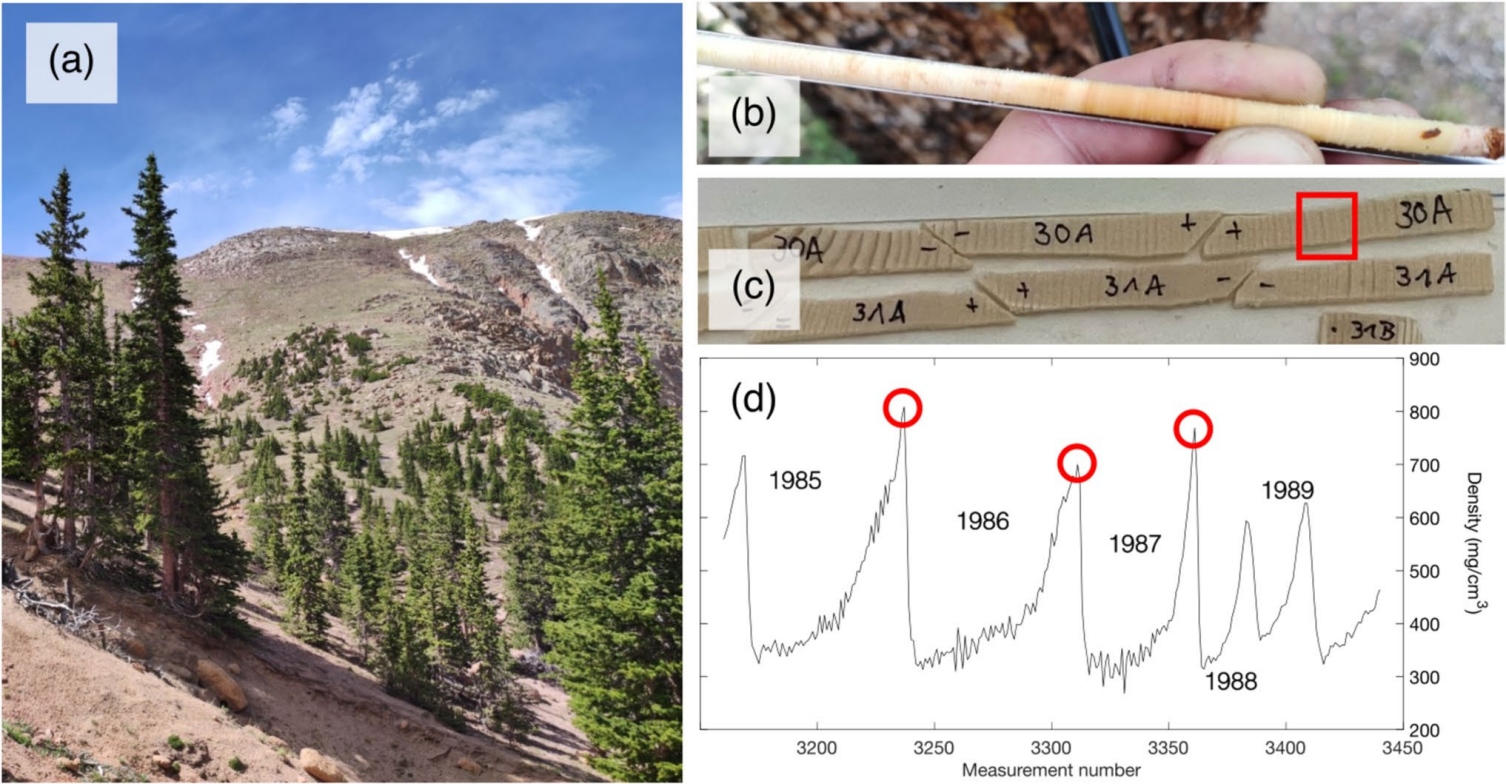
**a** Photograph of *Picea engelmannii* trees at the sampling site in Pike National Forest, Colorado. **b** Increment core sample from PNF. **c** Samples of wood laths prepared for X-ray densitometry. Red square indicates years 1985–1989. **d** Density profiles from five individual growth rings from sample PNF30A. Red circles indicate the MXD value for 1985, 1986, and 1987

In June of 2022, 58 Engelmann spruce trees were sampled for increment cores at Pike National Forest (PNF), Colorado (38.87°N, 105.07°W). Trees were located between 3,500 and 3,600 m.a.s.l., near the treeline on a northeast-facing slope (Fig. 1a). Two samples were collected from all trees, totaling 116 samples (Fig. 1b). Efforts were taken to ensure that trees from a wide range of ages were represented in the final dataset and included sampling trees in juvenile phases.

Increment cores were processed according to standard dendrochronological techniques (Stokes and Smiley 1968). Tree rings were assigned calendar dates through cross-dating, and their total-ring widths (TRW) measured. Density measurements from 113 of the samples were derived from X-ray densitometry using machinery from Walesch Electronics, following standard preparation procedures (Björklund et al. 2019). Cores were split into 1.02 mm thick laths, of 2–3 cm length, perpendicular to the longitudinal axis of the wood tracheids (Fig. 1c). The laths were exposed to X-rays for 14 min, and then compared to calibration materials, thus enabling the density of the wood to be established. For each core, several thousands of density measurements were produced, resulting in density profiles for each radial growth ring (Fig. 1d). A total of 827,554 density measurements were produced from 113 increment cores, and time series of the ring maximum density (MXD) for each processed core.

Individual series of TRW and MXD were standardized prior to averaging to produce site chronologies. The standardization included power transformation, detrending using an age-dependent curve spline (66% of length, with a 50-year starting point), and variance stabilization (Cook and Peters 1981), using the computer program ARSTAN v48d (Cook and Krusic 2005). The same approach was used for both TRW and MXD. The resulting standard chronologies were used for further climate comparison and reconstruction.

### Climate data and proxy signal identification

Monthly-resolved minimum (T_min_), mean (T_mean_), and maximum temperature (T_max_), as well as precipitation totals, were extracted from the gridded PRISM climate data network (Daly et al. 1994) for the period 1895–2021. The same climate parameters from CRU TS v4.06 (Harris et al. 2020) were analyzed for 1901–2021. Self-calibrated Palmer Drought Severity Index (scPDSI, Palmer 1965; Wells et al. 2004) data from CRU TS v4.07 (Harris et al. 2020) were also used, covering 1901–2021. Pearson’s correlations were calculated for the TRW and MXD chronologies against monthly and seasonally averaged PRISM/CRU climate data of the closest grid point for the longest overlapping period (1895/1901–2021). To test signal stability, correlation values for several sub-periods were also obtained and the statistical significance of differences were tested through a Fisher’s z-transformation test (Fisher 1921; Meko et al. 2011). Correlations with daily-resolved PRISM data (Daly et al. 2021) were calculated for 10–31 day windows for 1981–2020 at the closest 4 km gridpoint of the sampling site. Statistical significance and the magnitude of correlation inflation stemming from multiple comparisons were evaluated through the approach outlined by Torbenson et al. (2025), using the WeaGETS weather generator for synthetic time series of temperature and precipitation (Chen et al. 2010).

### Calibration and verification of reconstruction model

The average of aggregated August T_max_ data was calculated from PRISM grid coordinates: 36.05–39.15°N and 104.65–107.25°W. This average was used as the instrumental predictand in the reconstruction model. A simple linear regression makes up the calibration model for the reconstruction of August T_max_, for which the transfer function is:

$${rT}_{t}=(16.51*{X}_{t})+7.16$$

where rT is the reconstructed temperature and X is the MXD chronology value for year *t*. The calibration was performed on the longest possible period (1895–2021), as well as split periods (1895–1957 and 1958–2021). For the shorter calibrations, left out data were used for verification, which included calculations of cross-validation reduction of error (CVRE) and validation coefficient of efficiency (VCE; Cook et al. 1999). The full period calibration was repeated iteratively as measurement series dropped out from the chronology back in time. A quantile mapping correction (Robeson et al. 2020) was applied to the final time series, using the full period of overlap with the instrumental target data as a reference period, to scale the variance of the reconstruction. Uncertainty of the reconstruction estimates were estimated as the prediction interval (Olive 2007). Coherence between the most replicated version of the reconstruction and the instrumental temperature target at different periodicities was assessed through bootstrap simulation (Percival and Constantine 2006) using Welch’s overlapped averaged periodogram method (Welch 1967) for the period 1901–2021. The ability of the reconstruction to capture extremes were assessed through the Extreme Value Capture (EVC) test outlined by McCarroll et al. (2015). Qualitative comparisons against historical records and early instrumental measurements were also made.

### Additional timeseries analyses

Various analyses of the resulting reconstruction were performed. The spectral power of the reconstruction was assessed through periodograms, using a discrete Fourier transform (Bloomfield 2000). Running percentiles of various window-lengths were calculated for the full reconstruction. Similarly, distributions of moving 100-year periods were fitted to assess changes of extremes and means over times. The same was done for 30 year intervals, representing the current and past climatologies. A modified Mann–Kendall test (Hamel and Rao 1998) was applied to the reconstruction to assess long-term trend. The reconstruction was also compared to the North American Drought Atlas (NADA; Cook et al. 1999). In addition to correlations, anomalies for years of the most ten warmest and ten coldest reconstructed Augusts were compared to the gridded June–August PDSI estimates of the NADA. A selection of ten random years for the overlapping period (for which the MXD record and the NADA both have data; 1662–2005) were resampled 10,000 times at each gridpoint in a bootstrap approach. The 1st and 99th percentiles of the ten-year averages were used to assess significance of anomalies.

## Results

The chronology of tree-ring parameters from PNF spans 1539–2021, with a fairly even decrease in sample size back in time (Supplementary Fig. 1). The two chronologies are moderately but statistically significantly correlated with each other (r = 0.35 for 1662–2021; *p* < 0.001), and similar relationships are present for the TRW and MXD of individual series. It is worth noting that AR1 for the MXD chronology is lower (r = 0.14) than for the TRW chronology (r = 0.56), yet TRW is also statistically significantly correlated with the prior year’s MXD (r = 0.32 for 1662–2021; *p* < 0.001).

### Proxy-climate correlations

**Fig. 2:**
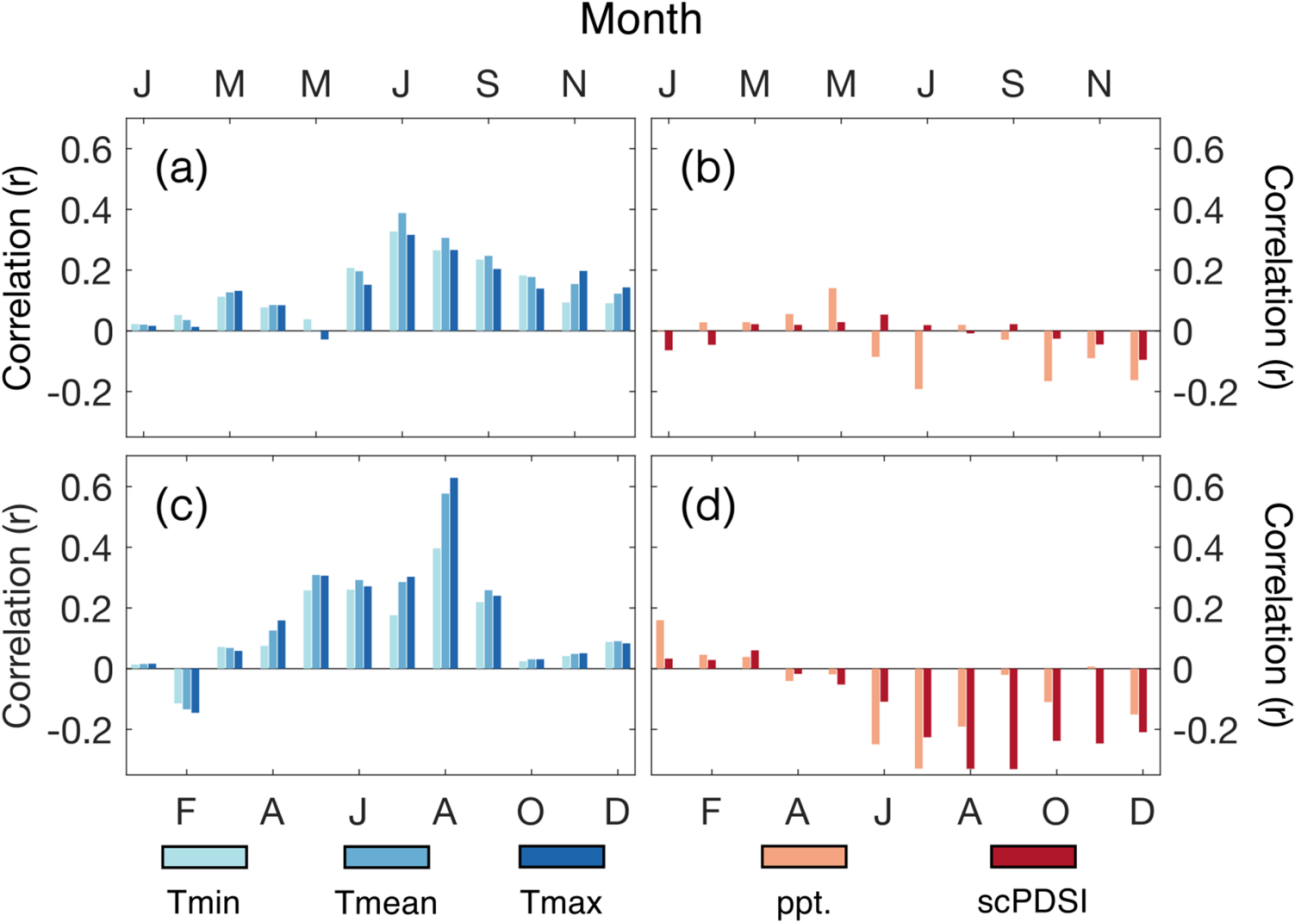
Pearson’s correlation coefficients for the TRW chronology and (**a**) temperature and (**b**) hydroclimatic variables of the closest PRISM gridpoint (closest CRU gridpoint for PDSI), for the longest overlap period (1895/1901–2021). (**c**) and (**d**): the same but for the MXD chronology

**Fig. 3:**
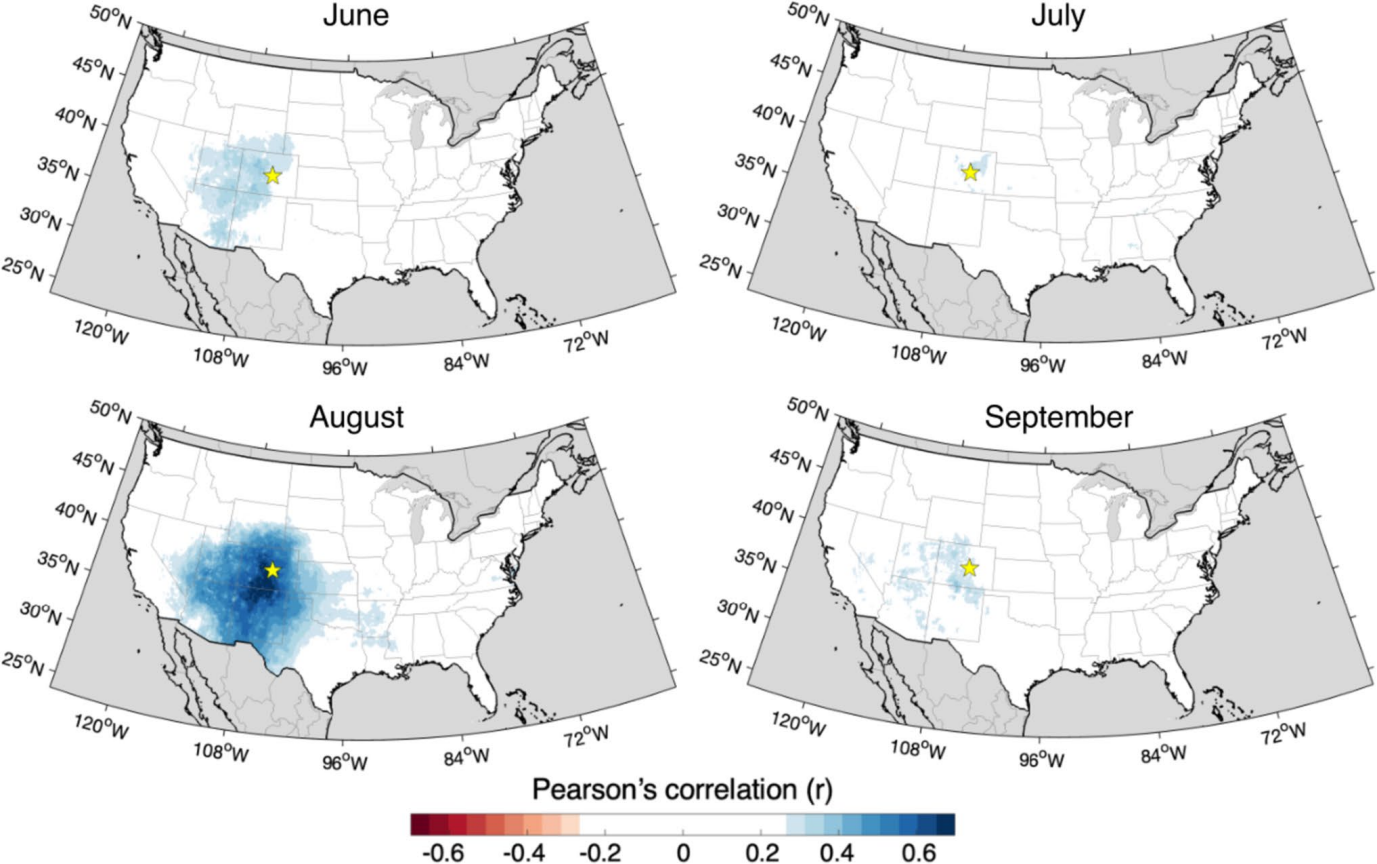
Mapped correlations between the MXD chronology and mean maximum temperatures for June, July, August, and September for the period 1895–2021. Colored correlations exceed r > 0.26. Yellow stars indicate the location of PNF

The TRW chronology displays its highest correlation with local monthly climate data (nearest PRISM gridpoint, 1895–2021) for July mean temperature (r = 0.38; *p* < 0.001) (Fig. 2a). The correlation strengthens marginally when allowing for a two-month average (July–August, r = 0.39; *p* < 0.001). The MXD chronology is most highly correlated with August T_max_ at r = 0.63 (Fig. 2c). This is the highest correlation obtained between MXD and T_max_, even when extending the climate window to include multiple consecutive months. T_max_ for May, June, July, and September also display statistically significant (*p* < 0.01) positive correlations (Fig. 3), as does T_mean_ and T_min_ for the summer months (Fig. 2c). However, the correlations with other temperature variables are all the strongest for single-month August. Little to no statistical significance is found in correlations between TRW and precipitation or scPDSI (Fig. 2b). Significant negative correlations are recorded between MXD and precipitation/scPDSI (r = −0.33 for August precipitation and September scPDSI; Fig. 2d), however, these correlation values are lower than the MXD-T_max_ values and weaken further when controlling for the relationship between temperature and hydroclimate (i.e., correlating the residuals with the MXD chronology; Meko et al. 2011).

Correlations with daily maximum temperature data were calculated for 10–31 day windows, with the ending Julian day starting at 213 (August 1st) and ending at 253 (September 10th) – resulting in 1312 possible windows/correlations. The highest correlation in this matrix is found for a 21-day window (Julian dates: 212–232), for which r = 0.744 (Supplementary Fig. 2). The difference between the correlation for this “optimum” window and the highest correlation found for the monthly resolved data (August) is, thus, r = 0.063. Simulations of pseudo tree-ring series and daily maximum temperatures from WeaGETS indicates that correlations will reach r = 0.767 by chance 1-in-20 times (an estimate of spuriousness; Torbenson et al. 2025) due to the high number of comparisons (Supplementary Fig. 3). The estimated *p* value, from the synthetic series, for the difference between the maximum correlations of daily and monthly climate data against the MXD chronology presented here is 0.148.

### Calibration and verification statistics

A simple linear regression calibration model (for 1895–2021) explains 52% of the variance in regional August T_max_ (Table 1; Supplementary Fig. 4a, b). Calibrating on the first half of the PRISM data produces strong verification statistics (validation reduction of error (VRE) = 0.52; VCE = 0.52; R^2^ = 0.52). Very similar statistics are present for the later period calibration (Table 1). Therefore, the signal in the MXD chronology is considered temporally stable and the reconstruction stemming from a calibration on the full 1895–2021 period is used in subsequent analyses. Results from the EVC test indicate that the reconstruction have skill in tracking both cold (*p* < 0.001) and warm (*p* < 0.01) extremes for the 127-year calibration period. R^2^ decreases as the number of available measurement series decline, but the available samples explain > 33% of the variance back to 1662 (Supplementary Fig. 4b). Of the twenty most extreme August T_max_ anomalies in the instrumental data (Supplementary Table 1), the reconstruction displays the same sign for eighteen of the years (eight for the cold, ten for the warm).

**Table 1:** Statistics of the August T_max_ reconstruction (CRE = calibration reduction of error, CE = coefficient of efficiency, RE = reduction of error)

	Calibration				Validation				
Period	r^2^		CRE		r^2^		CE		RE
1895–2021	0.52				/		/		/
1895–1957	0.53		0.53		0.53		0.52		0.52
1958–2021	0.53		0.53		0.53		0.52		0.52

The August T_max_ reconstruction displays coherency with the target data at all frequencies < 20-year periodicities (Supplementary Fig. 4c), indicating the MXD record’s ability to capture not only the interannual changes but also decadal-to-multidecadal variability. Few sources of historical information in the region exist prior to the calibration period but the discontinuous observational record for the period 1874–1888 (Diaz et al. 1982) has 1881 as the warmest August during this 15-year period – as does our T_max_ reconstruction.

### Variability of August maximum temperatures since 1662

**Fig. 4:**
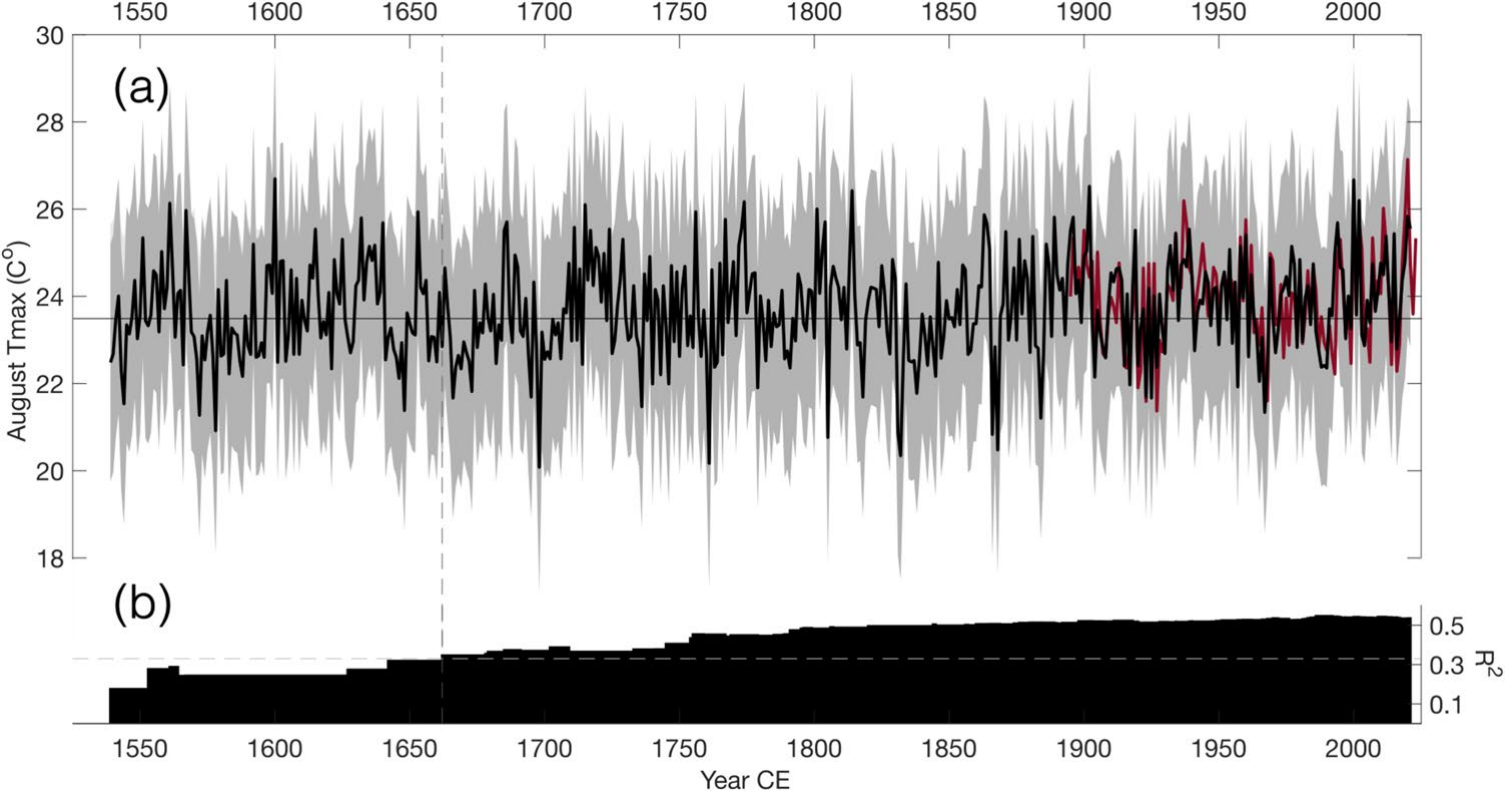
**a** Reconstruction of regional August T_max_ (black line) based on the maximum latewood density record, spanning 1539–2021. The dark red line represents the instrumental target data (1895–2023). Gray shading indicates the 95th percentile prediction interval. **b** Changing explained variance over time based on calibration statistics (for 1895–2021) for only trees/samples available at a given year. Dashed gray line indicates 33% explained variance, with a cutoff point of 1662 CE

The ten most extreme cold and warm Augusts since 1662, estimated from the density record, are presented in Table 2. The third coldest year is estimated for 1832, and is also part of short periods of consecutive negative anomalies (coldest two-year period: 1831–32; three-year period 1831–33), as well as the coldest decade (1831–1840; Fig. 4). A similar period is present in the earliest part of the reconstruction (1666–1675) for which nine of the ten years fall below the reconstruction mean. Considering the relatively low AR1 of the MXD record combined with the shared low-frequency variability of the predictor and predictand, these periods of negative temperature anomalies are likely reflecting true extended coolings. The warmest decade of the reconstruction is 1715–1724, followed by 1894–1903.

**Table 2:** The ten coldest and warmest years between 1662–2021 in the MXD-based August T_max_ reconstruction

Coldest		Warmest	
Year	C°	Year	C°
1698	20.08	2000	26.67
1761	20.17	1902	26.52
1832	20.34	1814	26.42
1868	20.47	2002	26.20
1805	20.76	1774	26.17
1866	20.83	1715	26.10
1831	20.87	1801	26.00
1884	21.20	1756	25.94
1967	21.33	1863	25.87
1736	21.47	2020	25.83

**Fig. 5:**
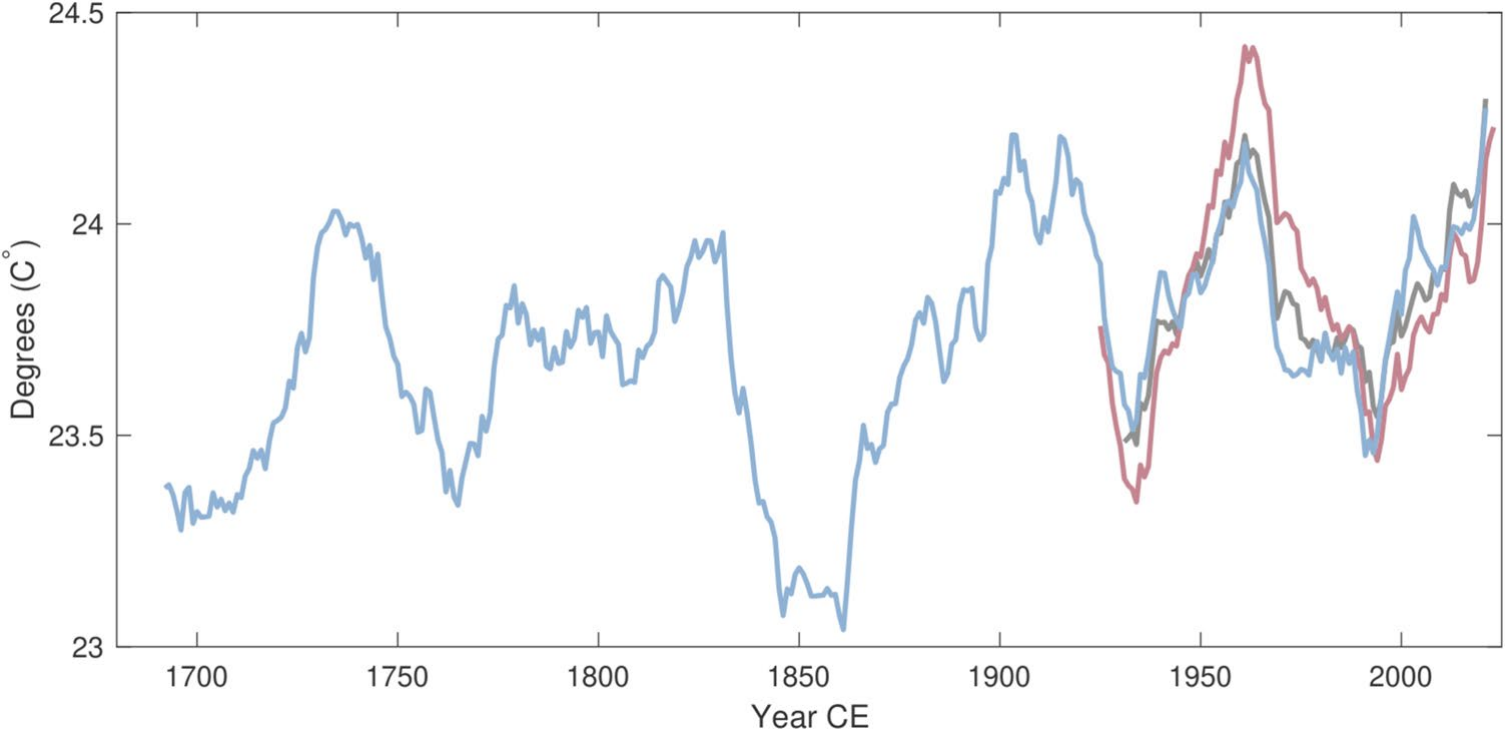
Running 30-year means (end year) of instrumental PRISM (red), CRU (black), and MXD-reconstructed (blue) August T_max_. The mean difference between CRU and PRISM has been subtracted from the CRU series

**Fig. 6:**
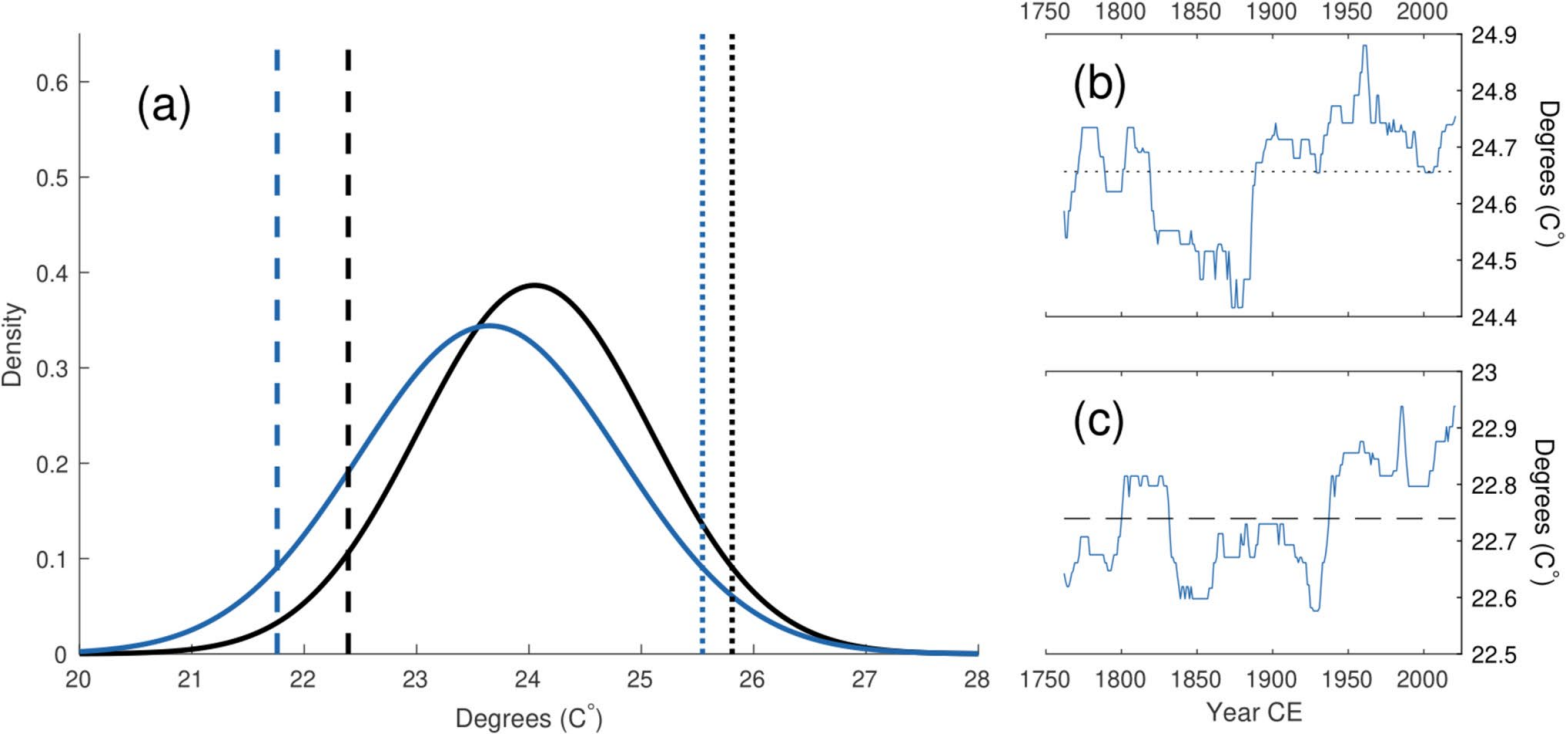
**a** Normal distributions fit to the reconstructed August T_max_ values for the pre-1969 period (blue) and post-1968 period (black). The 5th (dashed) and 95th (dotted) percentiles are plotted. Running 100-year 80th **b** and 20th **c** percentiles of the reconstructed August T_max_, plotted for end dates. Dotted/dashed lines indicate the respective 1662–2021 percentiles

The reconstruction displays positive and statistically significant (*p* = 0.001) trend for 1662–2021, with an average increase of 0.17 °C per hundred years. The most recent 30-year period also displays the highest average August T_max_ of the full reconstruction (Fig. 5). The trend for 1662–1900 is also positive and statistically significant (*p* = 0.036) but not for 1895–2021 (*p* = 0.504) or 1901–2021 (*p* = 0.106). For the instrumental regional average, no positive trends are recorded for 1895–2021 (PRISM *p* = 0.900) or 1901–2021 (PRISM *p* = 0.294; CRU *p* = 0.256). When limiting the analysis to estimates of the distribution tails, long-term changes are more pronounced for cool (< 20th percentile; Fig. 6c) than wam (> 80th percentile; Fig. 6b) anomalies.

## Discussion

Maximum latewood density variations in Engelmann spruce from Pike National Forest display a strong and stable relationship with local and regional August T_max_. This single-month signal is temporally stable across 127 years of instrumental data. The increase in instrumental temperatures recorded over recent decades is faithfully captured by the reconstruction, with average August T_max_ for 1992–2021 being the highest for any 30-year period since 1662 (Fig. 5). This most recent period also ranks first if the less replicated period 1539–1661 is included.

Temperature signals in tree-ring proxies are often confined to the growing season (although see Pederson et al. 2004; Chen et al. 2012), but the length of seasonal window varies greatly in some of the strongest and most commonly used chronologies from a global perspective (Wilson et al. 2007; Wilson et al. 2016). For reconstructions of northern Hemisphere temperature variability, which include many notable MXD records as predictor chronologies, traditional boreal summer (i.e., June to August) has been a preferred seasonal target (Stoffel et al. 2015; Büntgen et al. 2021). Although the season is partly a compromise to account for differing local climatologies and species-specific differences in response, averaging temperatures over multiple months often produces higher correlations than for any given single month (e.g., Büntgen et al. 2024). This is not the case for the MXD data from PNF. The defined response may partly stem from a shorter window of cambial activity for tree-line trees, as has been recorded for other spruce species (Rossi et al. 2008), combined with late-season temperature responses at lower latitude sites (Björklund et al. 2017). MXD is also closely related to latewood cell-wall thickness (Cuny et al. 2019), a process that may take 40–60 days to complete (Rossi et al. 2008) but the rate of which is positively correlated with temperature (Cuny et al. 2019).

Similar results to ours were recorded by Heeter et al. (2021a), for which the regional reconstruction targeted August–September maximum temperatures and in which the blue intensity (BI; McCarroll et al. 2002) data from PNF displayed the strongest correlations with August T_max_. Of course, this similarity is expected due to the shared location and the fact that BI is considered a substitute to MXD. Other BI records from different parts of the world have displayed similar signals, confined to the latter stages of the growing season (Peng et al. 2024).

### Extremes and trends in reconstructed August maximum temperatures

Only one of the 10 most extreme cold years estimated since 1662 have occurred since 1900 (Table 2), and none of the 30 coldest years is recorded in the past 30 years (1990 ranks as the 29th lowest August T_max_). The coldest August since 1900 in the reconstruction is 1967. Other notable low maximum temperatures are recorded for 1666, 1761, and 1698 – the latter representing the coldest temperature of the reconstruction. Conversely, three of the ten warmest years are recorded in the twenty-first century.

Extended periods of negative and positive temperature anomalies are also recorded throughout the reconstruction, similar to the twentieth century examples that the instrumental and reconstructed data share. The decadal stretches of pre-instrumental lows and highs (such as the cooler early 1700s and the 1830s, and the warmer 1710s-20 s and latter half of the 1800s) indicate that the low-frequency variability in the relatively short instrumental data reflects, at least partly, a real and considerable component of natural varaibility in August T_max_. Of such periods, 1931–1940 ranks as the third warmest decade in the reconstruction. The co-occurring Dust Bowl drought (Cook et al. 2009), perhaps the most severe climatic event in recent North American history, had significant impact on Colorado with agricultural failures and health problems (Hansen and Libecap 2004) leading to migration from the state (Worster 1979). Extreme heat is thought to have played a non-neglible role in the severe conditions that plagued the Great Plains for most of the 1930s (Donat et al. 2016). Although the average reconstructed temperatures for this decade is higher (24.71 °C) than the most recent ten years of the reconstruction (24.48 °C), 2012–2021 contains two years of greater temperatures than the highest estimated for the 1930s. When extending the temporal window beyond 10 years, the recent period stands out further, with the reconstruction suggesting that recent decades are the warmest of the full study period. Comparing temperatures prior to and after 1968 indicates a change of 0.4 °C (Fig. 6a). This increase appears driven, at least to some degree, by an upward shift in years of low T_max_ (Fig. 6c) when compared to changes in years of high temperatures (Fig. 6b).

Considerable differences in 30-year means for August T_max_ are observed between CRU and PRISM, with the instrumental data (PRISM) displaying higher average temperatures for the 1940s-60 s than for the most recent period. Such differences are present in other regions of the world (e.g. Büntgen et al. 2024) and the discrepancies highlight some of the uncertainties that still exist in gridded temperature products, even in more recent periods. Our reconstruction aligns better with the CRU data (Fig. 5), however, these differences may be due to differences in stations included and the exact spatial boundary of the regional averages. More importantly, there is significant warming over the full period of reconstruction. Although multicentennial-scale trend can be difficult to capture in tree-ring proxies (Cook et al. 1995), the agreement between predictor and predictand across all periodicities (Supplementary Fig. 4c) as well as the relatively even age-distribution (Supplementary Fig. 1) should lend credence to a possible slow but steady increase in August T_max_ over at least 350 years.

### Comparisons with other reconstructions

As expected, the MXD record presented here correlates highly (r = 0.70 for 1901–1983; and 0.72 for 1539–1900) with the local MXD collection from the early North American density network (Briffa et al. 1992). However, the non-shared variability appears, at least in part, to be related to strength of climate signal. The Briffa record displays a correlation of r = 0.46 with PRISM August T_max_ (for the longest possible overlap; 1895–1983) while PNF is correlated at r = 0.74 for the same period (difference significance of *p* = 0.001 in a Fisher’s z-transformation test; Snedecor and Cochran 1989). We argue that the difference is related to the elevation of trees sampled, for which the Briffa et al. MXD chronology are based on samples from 3,120 m.a.s.l. (some 450 m lower than the trees analyzed here). Distance to treeline, and its impact on temperature signal strength, has been studied in detail in western United States (Salzer et al. 2009). Trees of the same species, growing on the same slope, can not only lose climate sensitivity but even reverse its relationship with temperature over a 300 m elevational transect (Bunn et al. 2011). Similar elevation-related differences in the strength of temperature signal were also recorded in latewood BI data from the region (Heeter et al. 2021a).

The August–September T_max_ reconstructions based on BI records by Heeter et al. (2020; 2021a) for encompassing or nearby regions share many of the pre-twentieth century extreme years and multi-year periods in our reconstruction. These include the 1831–33 period, including the 3rd (1832) and 7th coldest (1831) Augusts in our estimates (Table 2), which also coincide with an extended period of low temperatures in millennia-long reconstructions from the southern Rockies based on *Pinus aristata* TRW chronologies (Salzer and Kipfmueller 2005). This anomaly is likely connected to a volcanic eruption, depositing sulphate in Greenland ice cores for 1831–1833 (Sigl et al. 2013). The 1830s continued with cooler temperatures, with estimates for 1835–1838 all falling below the 20th percentile of the reconstruction period (1662–2021). These years align with the 1835 Cosiguina eruption (Sigl et al. 2013), which has been linked to extensive cooling in northeastern United States (Harley et al. 2024) and northwestern Wyoming (Heeter et al. 2021b).

**Fig. 7:**
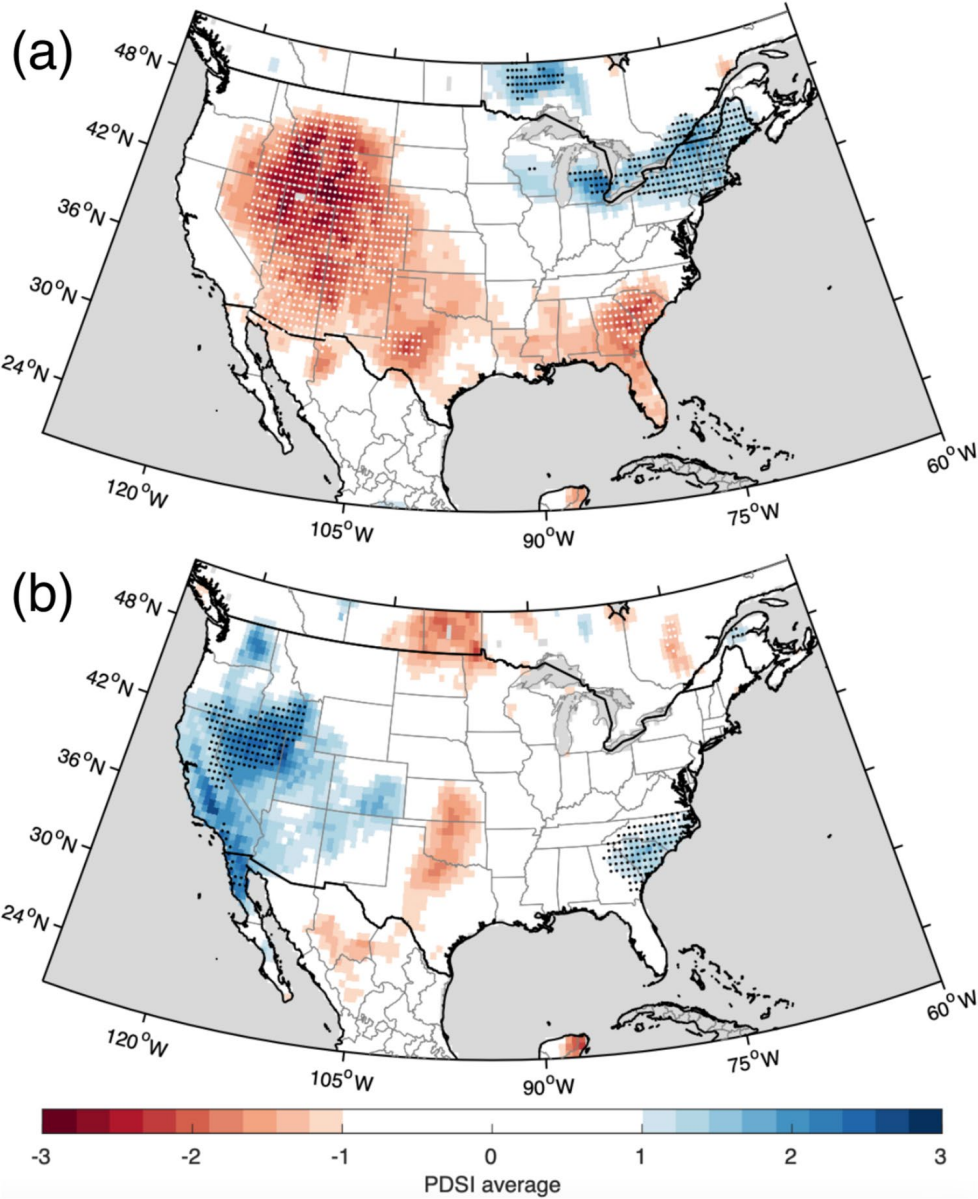
Average JJA PDSI anomaly from the North American Drought Atlas (Cook et al. 1999) for: (**a**) ten warmest years; and (**b**) ten coldest years in the August T_max_ reconstruction (1662–2005; Table 2). Dotted grid points indicate *p* < 0.01 for dry (white) and wet (black) conditions based on a 10,000-run bootstrap resampling at each individual grid point

The correlation between reconstructed August T_max_ and local instrumental JJA PDSI for 1901–2021 is r = −0.24 (−0.33 for August PDSI, Fig. 2; instrumental August T_max_ vs. August PDSI r = −0.21), and for the closest NADA grid point r = −0.16 for 1662–1900. The drought anomalies recorded by the NADA for much of western United States in the ten warmest year of reconstruction (Fig. 7a) indicate a strong relationship in years of extreme. None of these years (which include all of Table 2 with the exception for 2020 (not covered by the NADA) which is substituted for 1896, the 11th warmest year) fall during the Dust Bowl event, but, as described above, the 1930s stand out in terms of continuous departures of warm conditions in Colorado.

### The curious case of 1698

The correlation between TRW and MXD at PNF is (weakly) positive, comparable to tree-ring paramaters at many other high-elevation or high-latitude locations (Björklund et al. 2017; Hartl et al. 2022). Although the climate signal in the TRW record is weaker (Fig. 2a), this TRW-MXD relationship is likely to be driven by a shared temperature driver of variability. Considering the positive correlation, it is remarkable that the year of lowest reconstructed August T_max_ (and lowest MXD value of the chronology) cooccurs with the year of the widest ring for the period 1662–2021 (Supplementary Fig. 5). The coldest reconstructed value is, as noted, recorded for 1698. This year has been highlighted in previous studies (e.g., D’Arrigo and Jacoby 1999; Jones et al. 2005) and ranks high in the coldest summers in the Northern Hemisphere over the past 600 years by Briffa et al. (1998). It is the first year in a period thought to have occurred during the midst of several volcanic eruptions (Wiles et al. 2014), with possible sources in northwest North America (Clackett et al. 2018).

Increased radial growth during or following years of volcanic activity is not unprecedented in western North America. The best example is perhaps the extra-ordinary growth recorded for 1816 in Texas by moisture-sensitive tree-ring chronologies (Fye and Cleaveland 2001; Cleaveland et al. 2011). This positive effect may, partly, be explained by a lack of evapotranspiration demand as a result of cooler temperatures. The TRW chronology does, however, not show any significant correlation with hydroclimate during the instrumental period (Fig. 2b). Additionally, gridded reconstructions of PDSI (Supplementary Fig. 6) and precipitation (Stahle et al. 2020) indicate that 1698 was not extreme in terms of Colorado hydroclimate. Regardless of mechanisms and potential climatic drivers, 1698 stands out as a year of exceptional biological activity in Engelmann spruce in Colorado. Such examples of anomalous growth patterns deserve further study as they may inform our understanding of not only bioclimatic relationships but also mechanisms of biomass accumulation and carbon sequestration.

## Conclusions

We developed a MXD chronology from high-elevation Engelmann spruce in central Colorado. The climatic signal embedded in the density of the annual growth rings is confined to the month of August, with the MXD time series explaining half of the variance in maximum temperatures for a 127-year overlapping period with an instrumental regional average. The subsequent reconstruction shares both high- and low-frequency variability with observed summer temperatures. Extremes in the reconstructions include the 1830s, a period of volcanic activity. Conversely, August T_max_ in Colorado were less affected by larger and well-known eruptions, such as the 1815 Tambora event. A positive and statistically significant trend is present for the full reconstruction period, and recent decades represent the warmest 30-year periods over the past 350 + years. The current warming, possibly superposed on centennial-scale trend, appears to be driven to a greater extent by a lack of cold years, rather than a shift towards more extreme warm years.

## Electronic supplementary material

Below is the link to the electronic supplementary material.

ESM 1 (PNG 17 KB)

Supplementary file1 Figure 1. Distribution of individual core samples (gray) and trees (black) at any given time of the chronology. The dashed line indicates 1662 – the cutoff point for the analyzed reconstruction (TIFF 45 KB)

ESM 2 (PNG 139 KB)

Supplementary file2 Figure 2. Running correlations between the MXD chronology and daily-resolved Tmax for 10-31 day windows, for the period 1981-2020. Light orange shading indicates window of significance testing, darker orange highlights the window identified as the strongest monthly variable (TIFF 711 KB)

ESM 3 (PNG 51 KB)

Supplementary file3 Figure 3. Kernel-fitted density distributions of the difference between correlations of the MXD chronology with monthly August Tmax and daily-averaged Tmax of 1312 different Julian date windows, tested 10,000 times on 40 years of synthetic data (TIFF 67 KB)

ESM 4 (PNG 148 KB)

Supplementary file4 Figure 4. (a) Timeseries comparison between regional PRISM August Tmax (dashed line) and the MXD chronology, rescaled using the quantile mapping approach, regressed on the same temperature data (solid blue line). (b) Scatterplot between the same variables as in (a). (c) The squared coherence between instrumental data and MXD index for the period 1901-2021 is plotted (solid blue line), with dashed and dotted lines indicating the 95% and 99% confidence thresholds for statistical significance (TIFF 289 KB)

ESM 5 (PNG 65 KB)

Supplementary file5 Figure 5. Relationship between the MXD and TRW chronology from PNF. The year 1698 is highlighted (TIFF 106 KB)

ESM 6 (PNG 206 KB)

Supplementary file6 Figure 6. Reconstructed PDSI for 1698 (from the North American Drought Atlas; Cook et al. 1999) (TIFF 435 KB)

Supplementary file7 (DOCX 16 KB)

## Data Availability

The raw TRW and MXD data will be made publicly available through the International Tree Ring Data Bank (https://www.ncei.noaa.gov/products/paleoclimatology) upon publication.
