# Supplementary figures and images for: Lack of cold temperatures is driving recent high-summer warming in the southern Rocky Mountains
Source: Int J Biometeorol. 2025 Mar 31;69(6):1475–86. doi: 10.1007/s00484-025-02904-9 (PMC12141391; doi:10.1007/s00484-025-02904-9)

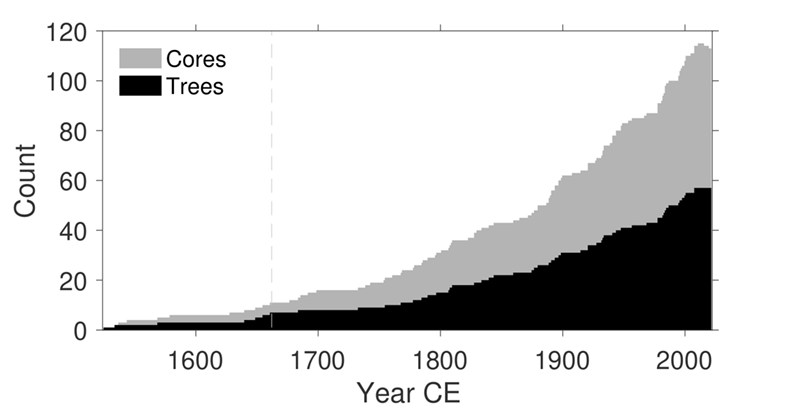

Supplement: Supplementary file 1 — (PNG 17 KB) [file 484_2025_2904_Fig8_ESM.png]

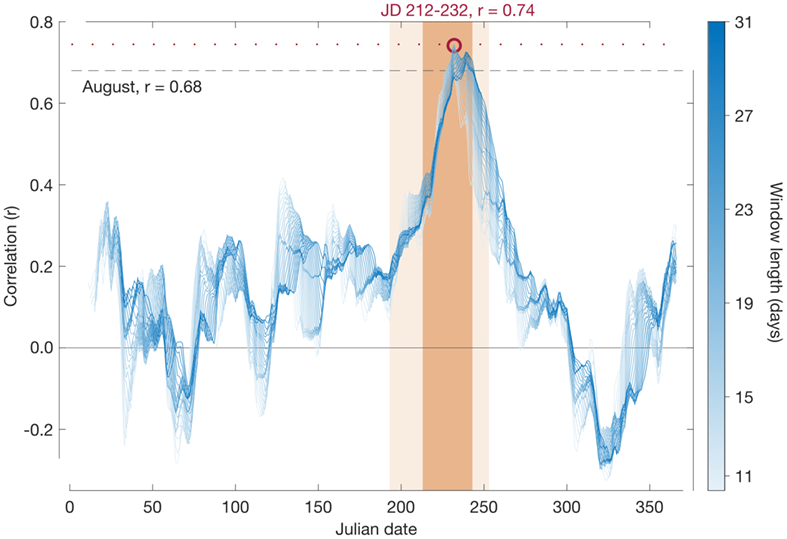

Supplement: Supplementary file 3 — (PNG 139 KB) [file 484_2025_2904_Fig9_ESM.png]

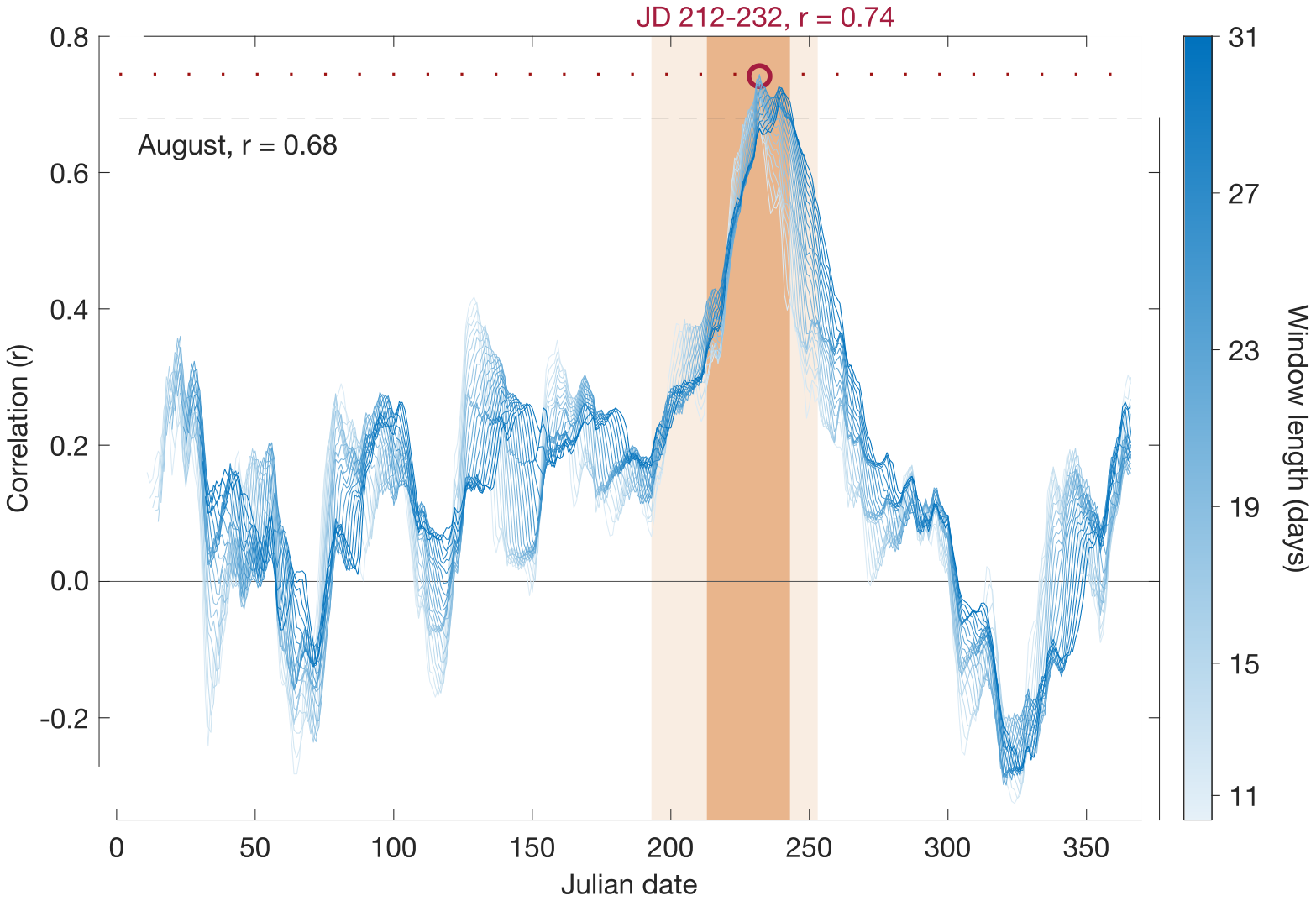

Supplement: Supplementary file 4 — Supplementary file2 Figure 2. Running correlations between the MXD chronology and daily-resolved Tmax for 10-31 day windows, for the period 1981-2020. Light orange shading indicates window of significance testing, darker orange highlights the window identified as the strongest monthly variable (TIFF 711 KB) [file 484_2025_2904_MOESM2_ESM.tiff]

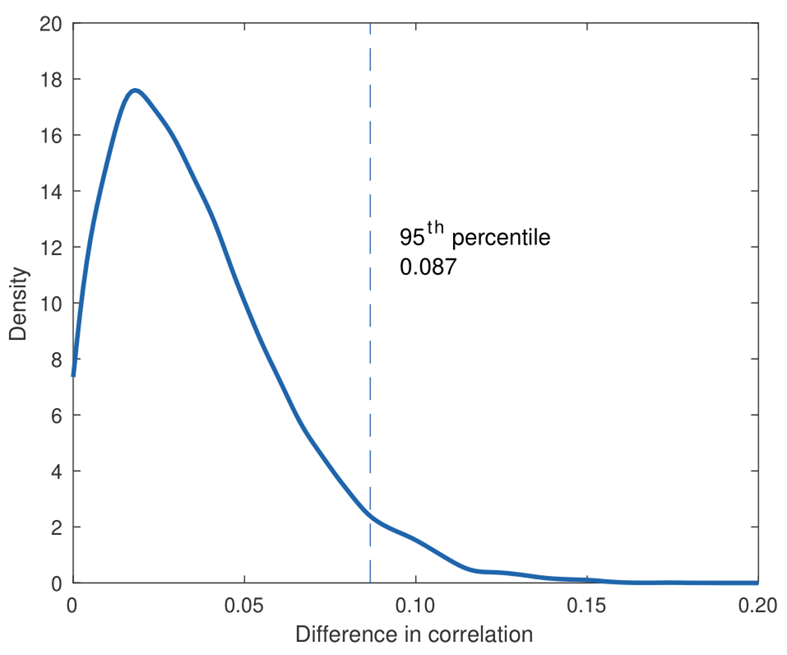

Supplement: Supplementary file 5 — (PNG 51 KB) [file 484_2025_2904_Fig10_ESM.png]

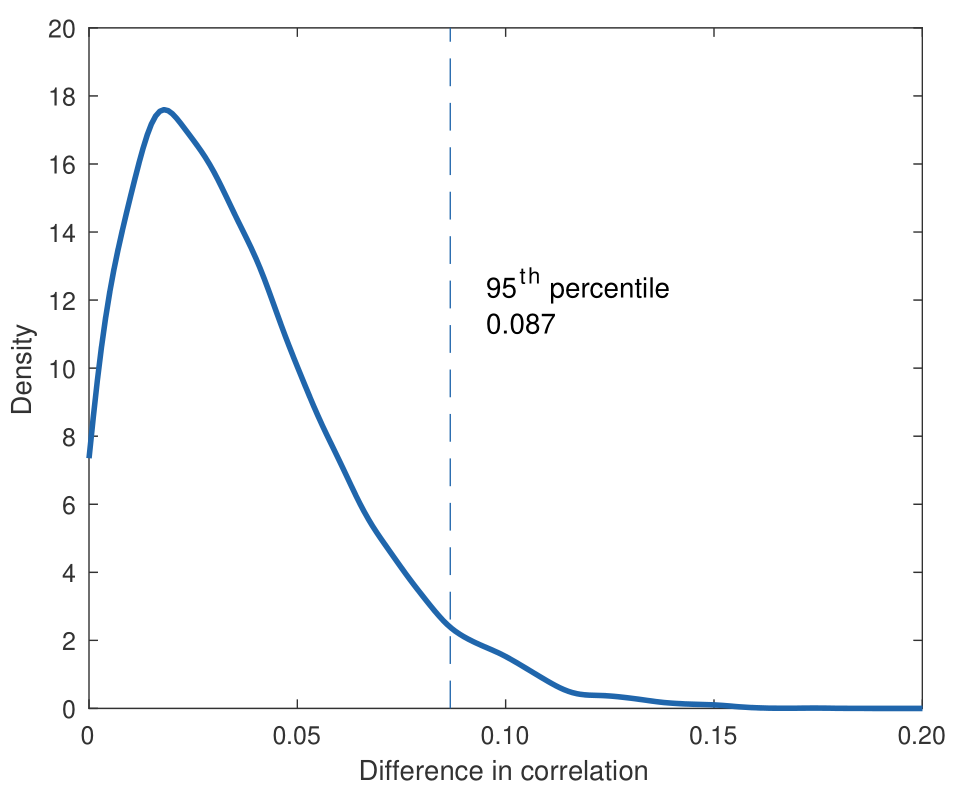

Supplement: Supplementary file 6 — Supplementary file3 Figure 3. Kernel-fitted density distributions of the difference between correlations of the MXD chronology with monthly August Tmax and daily-averaged Tmax of 1312 different Julian date windows, tested 10,000 times on 40 years of synthetic data (TIFF 67 KB) [file 484_2025_2904_MOESM3_ESM.tiff]

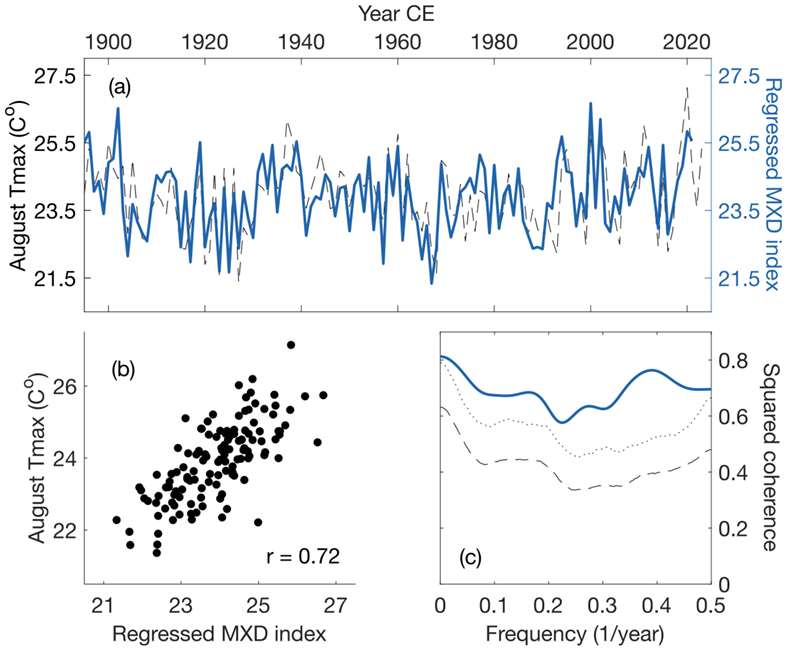

Supplement: Supplementary file 7 — (PNG 148 KB) [file 484_2025_2904_Fig11_ESM.png]

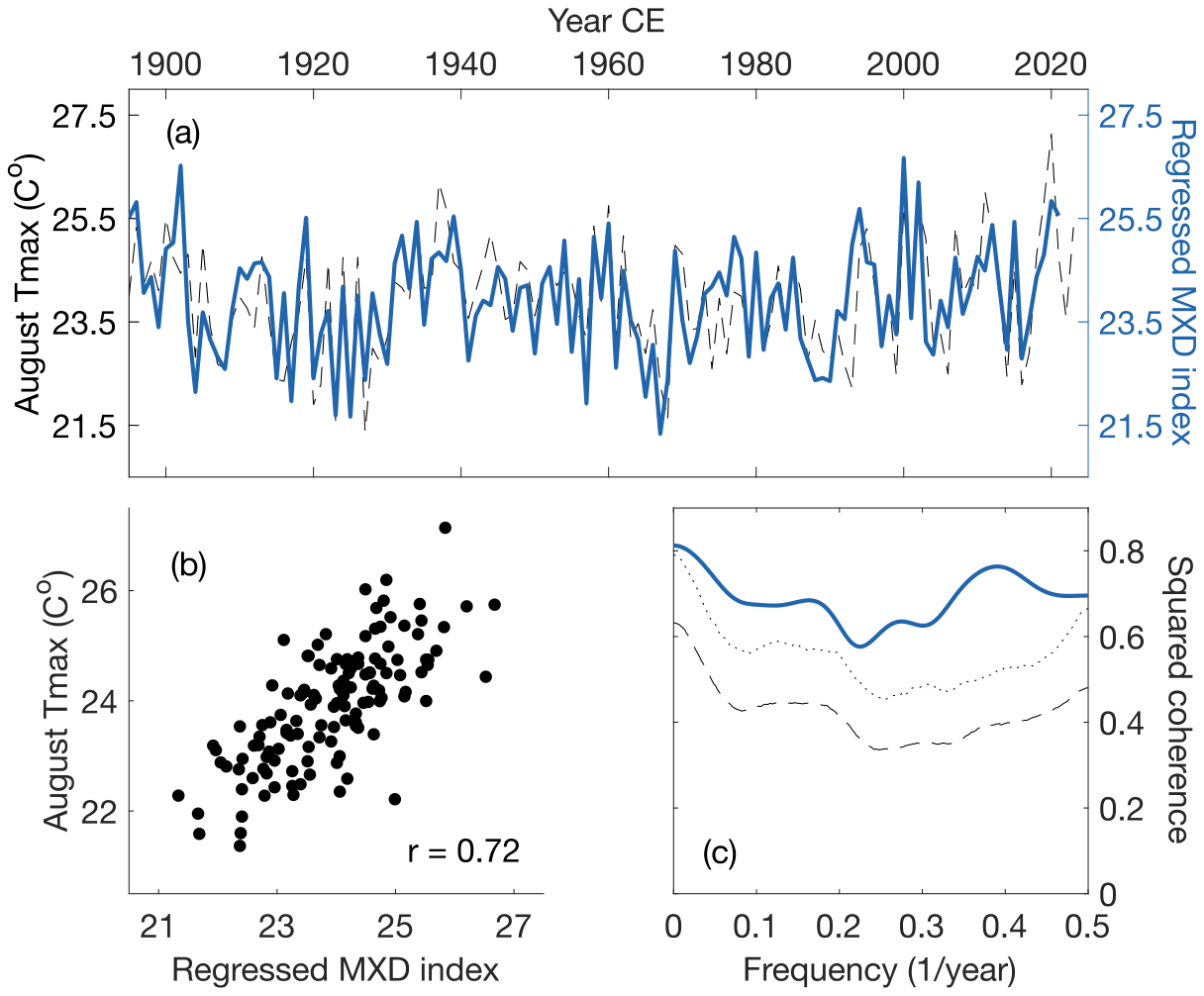

Supplement: Supplementary file 8 — Supplementary file4 Figure 4. (a) Timeseries comparison between regional PRISM August Tmax (dashed line) and the MXD chronology, rescaled using the quantile mapping approach, regressed on the same temperature data (solid blue line). (b) Scatterplot between the same variables as in (a). (c) The squared coherence between instrumental data and MXD index for the period 1901-2021 is plotted (solid blue line), with dashed and dotted lines indicating the 95% and 99% confidence thresholds for statistical significance (TIFF 289 KB) [file 484_2025_2904_MOESM4_ESM.tiff]

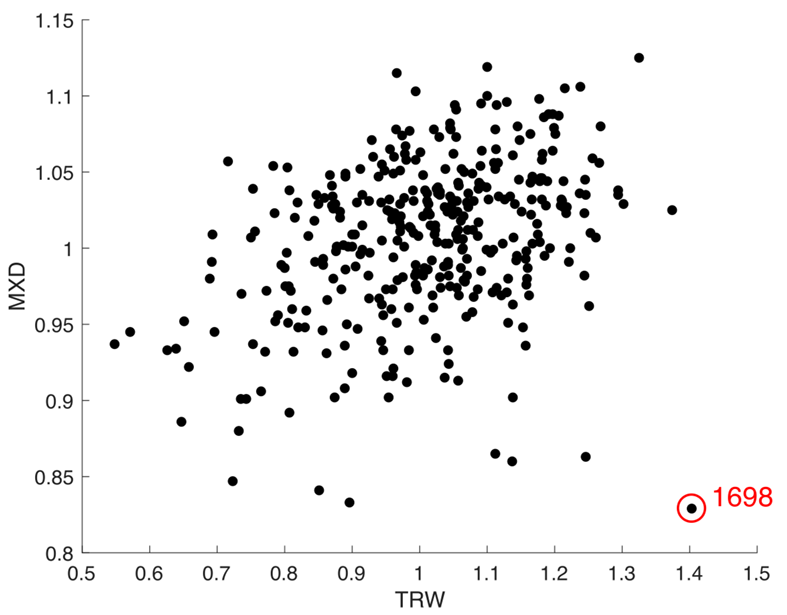

Supplement: Supplementary file 9 — (PNG 65 KB) [file 484_2025_2904_Fig12_ESM.png]

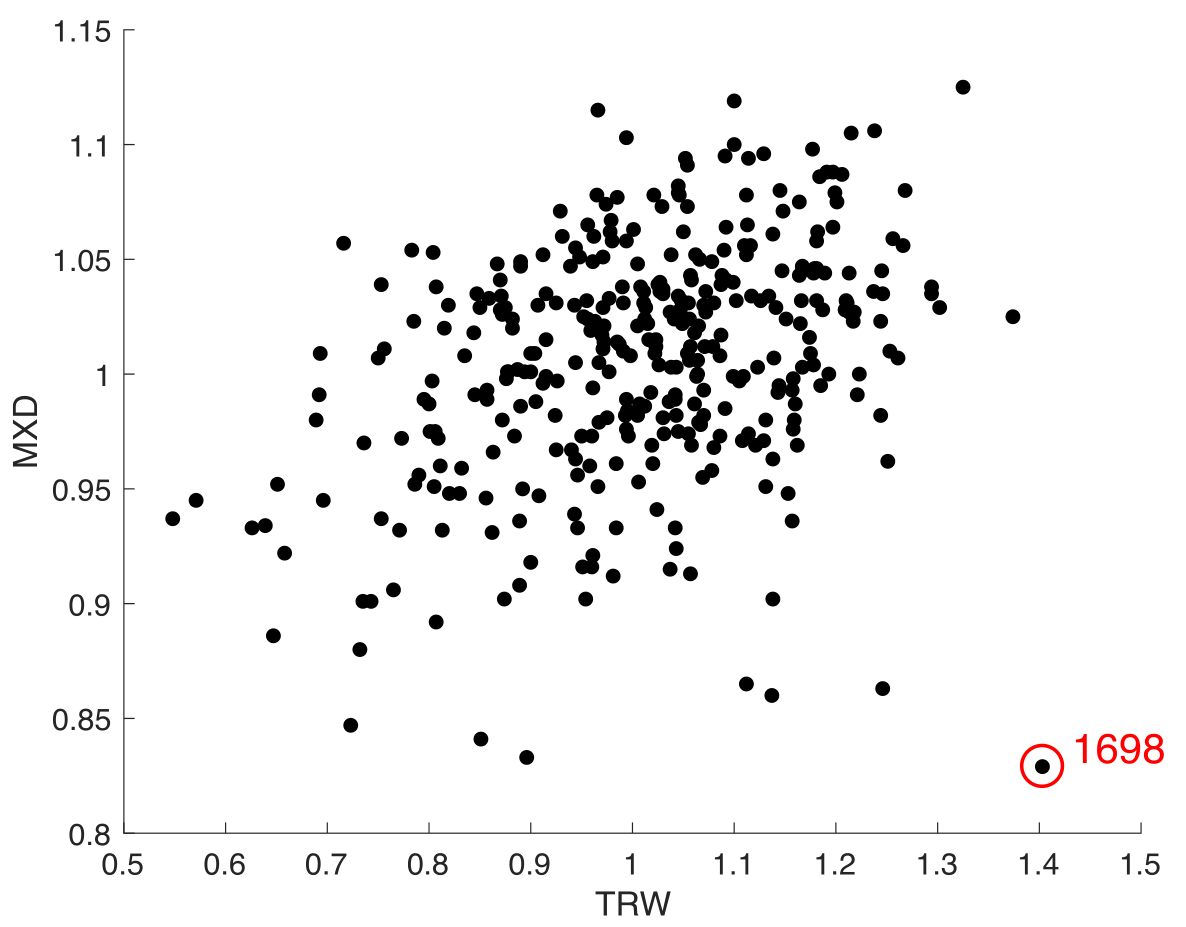

Supplement: Supplementary file 10 — Supplementary file5 Figure 5. Relationship between the MXD and TRW chronology from PNF. The year 1698 is highlighted (TIFF 106 KB) [file 484_2025_2904_MOESM5_ESM.tiff]

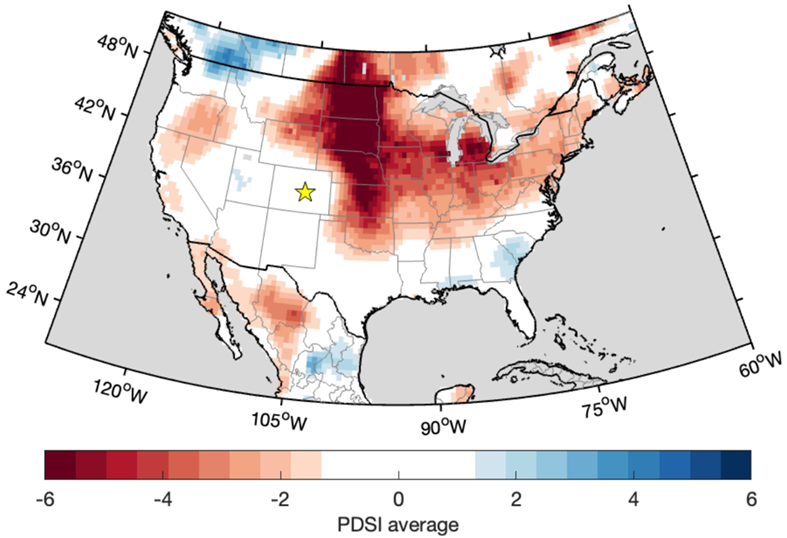

Supplement: Supplementary file 11 — (PNG 206 KB) [file 484_2025_2904_Fig13_ESM.png]

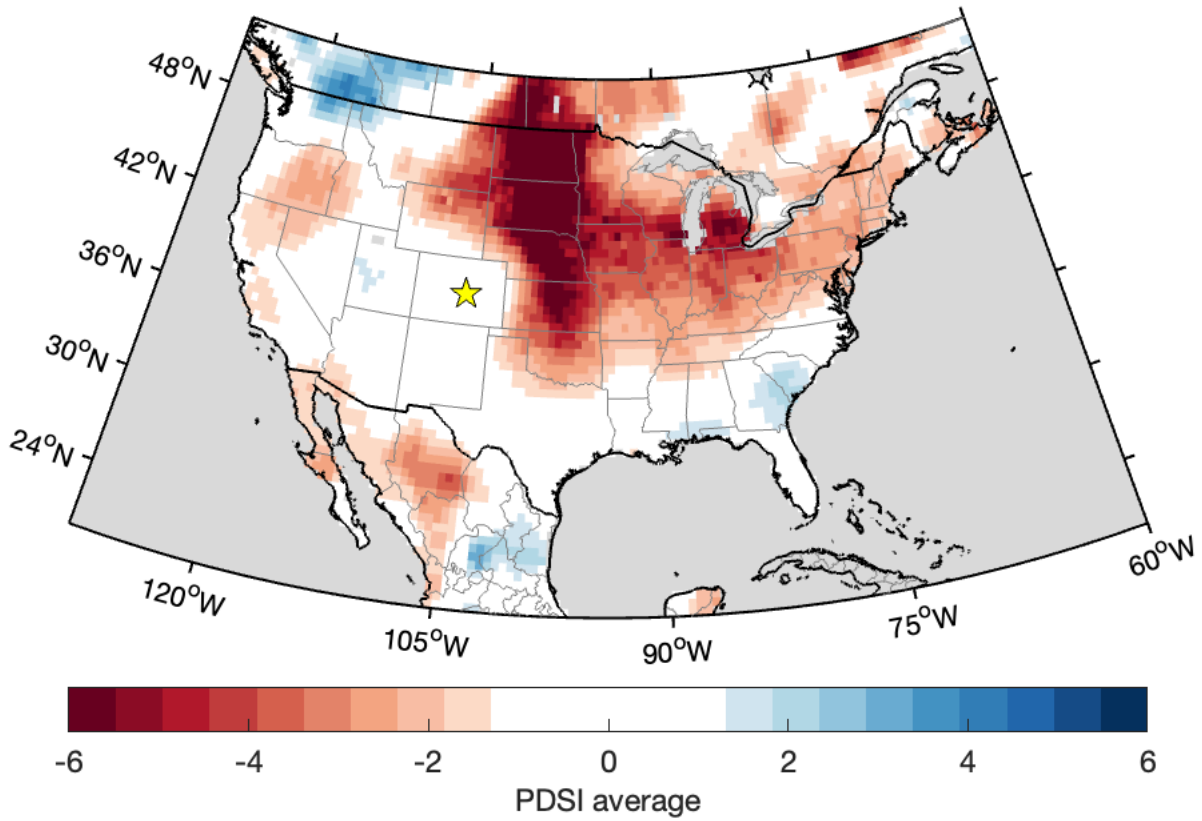

Supplement: Supplementary file 12 — Supplementary file6 Figure 6. Reconstructed PDSI for 1698 (from the North American Drought Atlas; Cook et al. 1999) (TIFF 435 KB) [file 484_2025_2904_MOESM6_ESM.tiff]
