# Supplementary material for: Lack of cold temperatures is driving recent high-summer warming in the southern Rocky Mountains
Source: Int J Biometeorol. 2025 Mar 31;69(6):1475–86. doi: 10.1007/s00484-025-02904-9 (PMC12141391; doi:10.1007/s00484-025-02904-9)
Supplement: Supplementary file 13 — Supplementary file7 (DOCX 16 KB) [file 484_2025_2904_MOESM7_ESM.docx]

**Supplementary Table 1**. The ten most extreme Z-score normalized August T_max_ values of instrumental (regional-averaged PRISM reconstruction target) and reconstructed data for the period 1895-2021.

| **Coldest** | | |  | **Warmest** | | |
| --- | --- | --- | --- | --- | --- | --- |
| **Year** | **PRISM** | **recon.** |  | **Year** | **PRISM** | **recon.** |
| 1927 | -2.36 | -1.44 |  | 2020 | 2.97 | 1.77 |
| 1923 | -2.16 | -2.07 |  | 1937 | 2.10 | 0.85 |
| 1968 | -2.15 | -1.42 |  | 2011 | 1.94 | 0.53 |
| 1920 | -1.87 | -1.40 |  | 2019 | 1.75 | 0.81 |
| 1925 | -1.82 | -2.09 |  | 1960 | 1.70 | 1.37 |
| 1993 | -1.58 | 0.99 |  | 2000 | 1.68 | 2.54 |
| 2016 | -1.52 | -1.05 |  | 2002 | 1.66 | 2.11 |
| 1967 | -1.52 | -2.40 |  | 1938 | 1.63 | 0.70 |
| 1921 | -1.51 | -0.60 |  | 1900 | 1.47 | 0.92 |
| 1916 | -1.45 | 0.13 |  | 1934 | 1.42 | 1.40 |
